# Supplementary material for: Longitudinal stability and interrelations between health behavior and subjective well-being in a follow-up of nine years
Source: PLoS One. 2021 Oct 29;16(10):e0259280. doi: 10.1371/journal.pone.0259280 (PMC8555827; doi:10.1371/journal.pone.0259280)
Supplement: S1 File — Data showing that omitting participants due to missing information on covariates does not cause major changes in the characteristics of the study population or distort the SEM model. (DOCX) [file pone.0259280.s001.docx]

**Supporting Information File 1: Comparison of the study population and respondents to the survey.** Data showing that omitting participants due to missing information on covariates does not cause major changes in the characteristics of the study population or distort the SEM model.

**Table A. Comparison between the study population and respondents of waves 2 and 3 according to gender and age. From Health and Social Support (HeSSup) study.**

|  |  | Study population (n=11,806)  % (n) | Respondents to waves 2 and 3 (n=13,050)  % (n) |
| --- | --- | --- | --- |
| Entire study population |  | 100 (11,806) | 100 (13,050) |
| Age | 25–29 | 20.7 (2,449) | 21.2 (2,768) |
|  | 35–39 | 20.5 (2,422) | 20.9 (2,723) |
|  | 45–49 | 26.7 (3,155) | 26.5 (3,461) |
|  | 55–59 | 32.0 (3,780) | 31.4 (4,098) |
| Gender | Male | 37.1 (4,382) | 37.6 (4,909) |
|  | Female | 62.9 (7,424) | 62.4 (8,141) |

**Table B. Path coefficient estimates in the crude structural equation model when based on the study population and on the entire sample of respondents to waves 2 and 3. From Health and Social Support (HeSSup) study.**

| Pathway |  | Study population (n=11,806) | Respondents to waves 2 and 3 (n=13,050) |
| --- | --- | --- | --- |
| Cross-sectional | HB03–SWB03 | 0.323 | 0.324 |
|  | HB12–SWB12 | 0.160 | 0.200 |
| Longitudinal | HB03–HB12 | 0.888 | 0.887 |
|  | SWB03–SWB12 | 0.471 | 0.473 |
| Cross-lagged | HB03–SWB12 | 0.150 | 0.151 |
|  | SWB03–HB12 | –0.005^a^ | –0.006^a^ |
| Observed items HB03 | Physical activity | 0.301 | 0.299 |
|  | Dietary habits | 0.430 | 0.428 |
|  | Alcohol consumption | 0.581 | 0.581 |
|  | Smoking status | 0.558 | 0.555 |
| Observed items HB12 | Physical activity | 0.372 | 0.373 |
|  | Dietary habits | 0.443 | 0.441 |
|  | Alcohol consumption | 0.568 | 0.577 |
|  | Smoking status | 0.576 | 0.576 |
| Observed items SWB03 | Happiness | 0.843 | 0.843 |
|  | Interest in life | 0.800 | 0.799 |
|  | Ease of living | 0.490 | 0.489 |
|  | Not feeling lonely | 0.640 | 0.639 |
| Observed items SWB03 | Happiness | 0.852 | 0.857 |
|  | Interest in life | 0.801 | 0.803 |
|  | Ease of living | 0.501 | 0.505 |
|  | Not feeling lonely | 0.645 | 0.647 |

HB03 = Health behavior in 2003

HB12 = Health behavior in 2012

SWB03 = Subjective well-being 2003

SWB12 = Subjective well-being 2012

^a^ non-significant (p > 0.05)
