# Supplementary material for: Longitudinal stability and interrelations between health behavior and subjective well-being in a follow-up of nine years
Source: PLoS One. 2021 Oct 29;16(10):e0259280. doi: 10.1371/journal.pone.0259280 (PMC8555827; doi:10.1371/journal.pone.0259280)
Supplement: S2 File — (DOCX) [file pone.0259280.s002.docx]

**Supporting Information File 2: Reliability measures**

Cronbach’s coefficient α is a commonly used measure of reliability. A required assumption for this to be a valid estimate is tau-equivalence, meaning that items have equal factor loadings. In a structural equation model where indicators of the same factor can have different factor loadings, tau-equivalence does not hold, and therefore Cronbach’s α might not necessarily be an accurate indicator of reliability.

A better estimate of reliability for congeneric models (i.e., different factor loadings) is the coefficient ω [1] usually attributed to McDonald [2]. For a factor with *k* continuous indicators, this coefficient can be calculated using the formula

$\omega=\frac{\left( \sum_{i} \lambda_{i} \right)^{2}}{\left( \sum_{i} \lambda_{i} \right)^{2}+\sum_{i} V\left( e_{i} \right)} ,$ *i*=1,..,*k*

where $\lambda_{i}$ is the factor loading of item *i* and $V\left( e_{i} \right)$ is the variance of the error in estimation of item *i*. However, the formula might not be applicable for factors with dichotomous indicators [3].

The values of Cronbach’s α for health behavior and subjective well-being were computed using SPSS v.27 and are given in Table A. Note that for binary data, Cronbach’s α coincides with Kuder-Richardson’s Formula 20, abbreviated KR-20. Also note that the assumption of tau-equivalence does not hold, as outlined above.

The values of McDonald’s ω were calculated using Mplus and are also given in Table A. As the health behavior indicators are dichotomous, no residual variances were obtained in the analysis, and therefore the coefficient ω could not be calculated properly in that case. The values of ω for health behavior were instead calculated when treating the indicators as being continuous, even though they are not so in the actual model.

**Table A. Reliability coefficients for health behavior and subjective well-being. From Health and Social Support (HeSSup) study.**

|  |  | KR-20 | Cronbach’s α | McDonald’s ω |
| --- | --- | --- | --- | --- |
| Health behavior | 2003 | 0.276 |  | 0.280 |
|  | 2012 | 0.303 |  | 0.309 |
| Subjective well-being | 2003 |  | 0.774 | 0.779 |
|  | 2012 |  | 0.784 | 0.790 |

**References**

1. Hayes AF, Coutts JJ. Use Omega Rather than Cronbach’s Alpha for Estimating Reliability. But…. Commun Methods Meas. 2020;14: 1–24. doi:10.1080/19312458.2020.1718629

2. McDonald RP. Test Theory: A Unified Treatment. Mahwah, NJ: Lawrence Erlbaum; 1999.

3. Raykov T. Reliability - binary and ordinal items. In: Mplus FAQs [Internet]. [cited 1 Jul 2021]. Available: https://www.statmodel.com/download/binary and ordinal items.pdf
